# ROOTS: An Algorithm to Generate Biologically Realistic Cortical Axons and an Application to Electroceutical Modeling

**DOI:** 10.3389/fncom.2020.00013

**Published:** 2020-02-21

**Authors:** Clayton S. Bingham, Adam Mergenthal, Jean-Marie C. Bouteiller, Dong Song, Gianluca Lazzi, Theodore W. Berger

**Affiliations:** ^1^Department of Biomedical Engineering, Case Western Reserve University, Cleveland, OH, United States; ^2^Department of Biomedical Engineering, University of Southern California, Los Angeles, CA, United States; ^3^Department of Electrical Engineering, University of Southern California, Los Angeles, CA, United States

**Keywords:** deep brain stimulation (DBS), axons, multi-scale, electrical stimulation (ES), morphology, spatio-temporal analysis

## Abstract

Advances in computation and neuronal modeling have enabled the study of entire neural tissue systems with an impressive degree of biological realism. These efforts have focused largely on modeling dendrites and somas while largely neglecting axons. The need for biologically realistic explicit axonal models is particularly clear for applications involving clinical and therapeutic electrical stimulation because axons are generally more excitable than other neuroanatomical subunits. While many modeling efforts can rely on existing repositories of reconstructed dendritic/somatic morphologies to study real cells or to estimate parameters for a generative model, such datasets for axons are scarce and incomplete. Those that do exist may still be insufficient to build accurate models because the increased geometric variability of axons demands a proportional increase in data. To address this need, a Ruled-Optimum Ordered Tree System (ROOTS) was developed that extends the capability of neuronal morphology generative methods to include highly branched cortical axon terminal arbors. Further, this study presents and explores a clear use-case for such models in the prediction of cortical tissue response to externally applied electric fields. The results presented herein comprise (i) a quantitative and qualitative analysis of the generative algorithm proposed, (ii) a comparison of generated fibers with those observed in histological studies, (iii) a study of the requisite spatial and morphological complexity of axonal arbors for accurate prediction of neuronal response to extracellular electrical stimulation, and (iv) an extracellular electrical stimulation strength–duration analysis to explore probable thresholds of excitation of the dentate perforant path under controlled conditions. ROOTS demonstrates a superior ability to capture biological realism in model fibers, allowing improved accuracy in predicting the impact that microscale structures and branching patterns have on spatiotemporal patterns of activity in the presence of extracellular electric fields.

## Introduction

**FIGURE 1:**
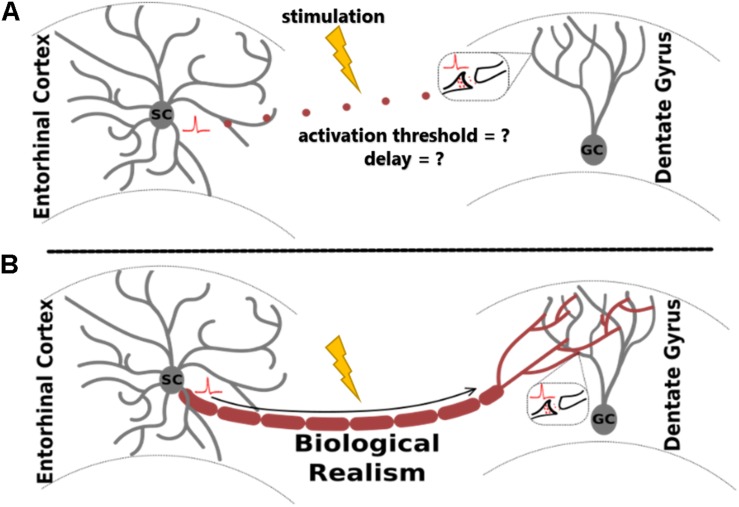
Adding biological realism to axon models for the study of extracellular electrical stimulation allows more accurate analysis of evoked neural network activity. The proposed algorithm, ROOTS, was developed specifically to provide increased realism in fiber models. The utility of ROOTS is to mitigate the challenge portrayed above: without explicit axonal reconstructions, how does one accurately estimate the site of action potential initiation and appropriate orthodromic conduction latencies through the terminal region in the presence of externally applied electric fields? **(A)** corresponds to the case where distance-based pure-delay mechanisms are used. **(B)** corresponds to explicit and biologically accurate axon representations.

In the study of extracellular electrical stimulation of neural systems, spatial and temporal patterns of activity are strongly influenced by tissue geometry. One established approach to studying this relationship is through morphologically detailed equivalent circuit models of neurons, including axons. While these models are invaluable for many different applications, they are especially useful for prediction of tissue response to extracellular stimulation, where explicit morphologies aid the prediction of activation thresholds under varying biological and stimulating conditions (Ranck, 1975; Nowak and Bullier, 1996, 1998). For biologically realistic network models, a common method involves arranging individual neuron models in virtual space to reconstruct elements of the tissue system being studied (Grill, 1999; McIntyre and Grill, 1999; Howell and McIntyre, 2016; Anderson et al., 2018; Bingham et al., 2018). This approach enables accurate prediction of membrane potentials in response to changes in electric field geometry and gradient (Clark and Plonsey, 1970; Figure 1).

Despite an understanding that geometry and topology influence activity, much of the biological realism in these studies is reserved for dendritic rather than axonal arbors. This lack of realism in axonal fibers becomes especially disconcerting when considering both that (i) central nervous system axon terminal arbors are often highly branched and tortuous relative to the mostly straight and long nerves of the periphery, and (ii) under most typical stimulating conditions, axons have shorter chronaxies than somas and dendrites (Ranck, 1975; Johnson and McIntyre, 2008; Rattay et al., 2012). It follows that suprathreshold stimulation events result in coupled local (driven by the injected electric field) and distal (synaptically driven) activity, with substantial realism being necessary to predict the spatiotemporal pattern of the resulting response in totality. Deliberate arrangement of neuronal structures is also useful for accurate model-based prediction of tissue–tissue interactions due to electric fields arising from endogenous current sources (Anastassiou and Koch, 2015). Accurate estimation of local field potentials (LFPs) and, therefore, predictions of the region-specific impact of ephaptic coupling are sensitive to the degree of biological realism implemented in a model system (Bingham et al., 2018). Lastly, network models that lack explicit axonal structures may have unrealistic conduction delays between connected populations of neurons, leading to potential prediction errors (Kim et al., 2019). While delays may be trivially added to network connections once they are known, biologically appropriate behaviors must first be estimated. Therefore, geometrical and anatomical realism may also be necessary to study emergent network activity such as co-oscillatory activity in hippocampal networks (Whittington et al., 1997; Fries, 2005).

Despite the long record of hippocampal observation, axonal morphology is not as scrupulously described as the somas and dendrites of many cell types. With a few exceptions, studies yielding explicit reconstructions through morphometric analysis of neuronal branching have focused on dendritic arbors and overlooked their axons (Desmond and Levy, 1985; Hama et al., 1989; Claiborne et al., 1990). The dearth of robust datasets is exacerbated by the general observation from staining experiments performed in the hippocampus that axonal structures, even of the same cell type, may be less stereotyped than dendritic arbors (Hjorth-Simonsen and Jeune, 1972). Perforant path axon terminal arbors from layer 2/3 entorhinal cortical (EC) spiny stellate cells are not constrained to simple conical, fanned, or star-shaped volumes like so many dendritic arbors (Tamamaki and Nojyo, 1993). Geometric and topological heterogeneity make the prospect of using explicit reconstructions unfeasible for direct use in computational models which require *in situ* cell density at tissue scale. This becomes particularly apparent when considering that the unique geometry of the dentate gyrus, which changes from septal to temporal poles, requires dramatic inter-region variety in the volume, orientation, and contour of afferent EC axons.

Despite the general absence of morphometrics for axons, the shape of terminal fields, distribution of synaptic spines, and a rough measure of anatomic domains of axon terminal fields from either histology or other imaging methods provide data from which minimally functional axons can be grown. Explicit dendritic reconstructions, spine counting, and anterograde and retrograde staining experiments provide information regarding synaptic targets. Distributions of synaptic targets combined with a knowledge of the general path and origin of a fiber is sufficient to generate a functional structure that captures the tertiary conformation and local divergence of the axon terminal arbor. When representing a neural process as a graph, nodes placed at synapses and edges to connect between them and the soma can effectively reconstruct a functional dendritic arbor (Türetken et al., 2011). Likewise, presynaptic boutons provide nodes that can be connected to each other and the soma with a deliberate arrangement of edges to form a spatial network or graph. In other words, a minimally functional axon connects a parent cell to synaptic targets.

Beyond minimally functional morphologies, sophisticated models of realistic neuronal branching have been proposed with primary application to either generating unique and artificial dendritic trees or reconstructing them from a series of images. There are two chief types of generative models: (i) stochastic (Rozenberg and Salomaa, 1980) or (ii) greedy (Cuntz et al., 2007, 2010; Budd et al., 2010; Budd and Kisvarday, 2012). Stochastic models operate by sampling distributions of branching statistics extracted from experimentally measured neurons, while greedy graph-based models are much more frequently used to reconstruct three-dimensional dendritic trees from stacks of manually labeled images (Türetken et al., 2011). Although each of these approaches have been useful under certain conditions, neither has been properly adapted for use in the generation of virtual biologically realistic axons.

Consequently, we present in this paper a new graph-based algorithm for generating biologically realistic tree representations of axon terminal arbors. The proposed model is inspected for its utility in studying extracellular electrical stimulation of cortical tissue through analysis of the impact of arbor topography and morphometry on activation thresholds and, by extension, spatiotemporal patterns of activity in the hippocampus. The results of this work comprise (i) a quantitative analysis of the generative algorithm proposed, (ii) presentation and quantitative/qualitative description of generated fibers, (iii) comparison to leading alternative methods, (iv) demonstration of a method to reduce spatial complexity of axonal arbors while maintaining accurate prediction of neuronal response to extracellular electrical stimulation, and (v) an extracellular electrical stimulation strength–duration study. The value of these studies is twofold: (i) establishing the novelty and utility that this modeling system yields and (ii) determining if stimulation–response recruitment order (i.e., large before small) for straight, long, large-diameter, and myelinated peripheral fibers is similarly true of small, highly branched, and unmyelinated cortical fibers.

## Materials and Methods

The study presented here focuses on accurately capturing the emergent spatial features of spiny stellate EC axons within the dentate gyrus in Sprague-Dawley rats. The model is designed to be flexible to the inclusion of novel morphometric criteria as new experimental data become available but, at present, it is clearly important that (i) fibers are constrained to 1–1.5 mm within the septotemporal axis (Tamamaki and Nojyo, 1993), (ii) laminar organization along the transverse axis is inviolate, with medial and lateral EC axons confined to the middle and outer thirds of the dentate molecular layer, respectively, and (iii) axons synapse with a pronounced *en passant* connective schema, where most pre-synaptic boutons are non-terminal. Many more features and their sources are detailed in Table 1. A greedy, graph-based, or ruled-optimum ordered tree system (ROOTS) was developed to control these features. First, we will explain the development of constraints and inputs to the method and then explain, in detail the functions of the method.

**TABLE 1:** Principle features **(left)** of entorhinal cortical axons found in the dentate gyrus perforant path and the studies which reported them **(right)**.

**Feature**	**References**
Strictly laminar dentate perforant path	Hjorth-Simonsen and Jeune, 1972; Witter, 2007
En passant; mostly non-terminal boutons	Witter, 2007; Bindocci et al., 2017
Distribution of bifurcation angles ≈80.3 ± 35.7°	Budd and Kisvarday, 2012
0.1 μm fiber diameter, ≈0.7 μm boutons	Tamamaki and Nojyo, 1993
Primary bifurcation at/near crest, envelopes entire transverse of dentate, continues to CA3	Hjorth-Simonsen and Jeune, 1972; Schwartz and Coleman, 1981; Tamamaki and Nojyo, 1993; Witter, 2007
≈17,700 synapses per EC axon	Desmond and Levy, 1985; Hama et al., 1989; Claiborne et al., 1990
Myelination – mixed, though clearest images show no myelination below the crest of dentate	Tamamaki and Nojyo, 1993

*These features, when combined with the topography of spines in the outer and middle thirds of the dentate gyrus provide guiding parameters for construction of artificial tree models. The proposed method utilized these as criteria for generation of dentate perforant path fibers that provide a test-case for ROOTS as a method.*

### Volume and Nodal Constraints

**FIGURE 2:**
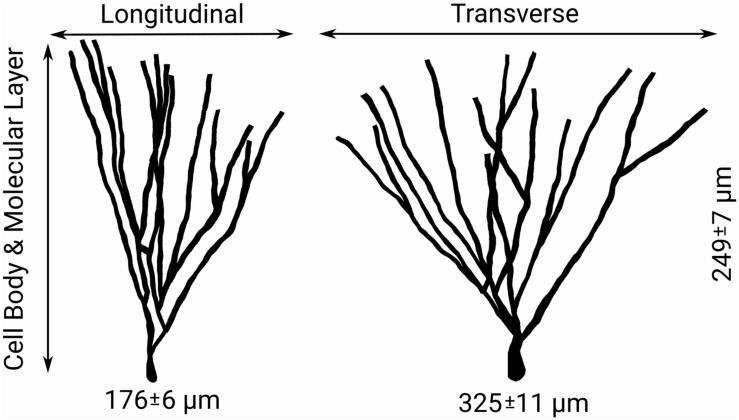
Features of granule cell arbors – including the distributions of longitudinal, transverse, and dentate normal lengths of arbors shown above – and afferent connectivity provides guiding information for both the sampling of target nodes for generation of axon morphologies and the simplification of generated morphologies (Claiborne et al., 1990).

In the construction of a spatial ordered tree, the number and topography of target nodes strongly determines the emergent features of the resulting graph. Therefore, the selection of nodes is an important step in the successful generation of biologically appropriate axonal trees. This process was executed using the Kjoenigsen rat hippocampal atlas slice at −3.34 from Bregma to segment model boundaries (Kjonigsen et al., 2008). Past efforts have elucidated the approximate number and spatial distribution of synaptic targets within the dentate perforant path (Claiborne et al., 1990; Bingham et al., 2016; Hendrickson et al., 2016). The approximate number and density of granule cells found within the dentate region of a 1.5 mm extruded slice was calculated based on density measurements reported in the literature (Gaarskjaer, 1978; Patton and McNaughton, 1995). Spine counts and the laminar topology of entorhinal–dentate connections were used to create a pool of synaptic targets and the number of perforant path arbors from which afferent connections might be formed (Desmond and Levy, 1985; Hama et al., 1989; Claiborne et al., 1990). An abbreviated table derived from Hendrickson et al. (2016) can be found below to summarize these parameters as they were used in this study:

To summarize: the number of synaptic spines in the outer and middle third of hippocampal granule cell dendritic arbors the size, number, and density of granule cells, and the number/density of EC cells contributing to the perforant path provide the necessary arithmetic for deducing the number of synapses made within each axon terminal field (Table 2 and Figure 2). When combined with observational data regarding the septotemporal range of these axons, this information provided an approximate volume throughout which nodes could be distributed and a plausible synthetic terminal arbor could be grown. These same parameters were used to construct much larger, more complex, and previously validated mechanistic models of a rat dentate hippocampus; therefore, additional details can be found in Bingham et al. (2018) and Hendrickson et al. (2016).

**TABLE 2:** Parameters describing the entorhinal cortical–dentate gyrus topology used to design the topography of synaptic targets for axon fiber growing described in later sections.

**Entorhinal cortical–dentate gyrus topological parameters**	
Granule cell # spines: middle 1/3:	1050–1200
Granule cell # spines: outer 1/3:	1100–1300

*This table provides a range of spine-counts per dentate granule cell with which the perforant path fibers may synapse (Claiborne et al., 1990; Desmond and Levy, 1985). Medial entorhinal cortical fibers synapse in the middle 1/3 and lateral entorhinal cortical fibers synapse in the outer third of the granule cell dendritic arbors.*

### Constraining Patterns of Axon Branching

**FIGURE 3:**
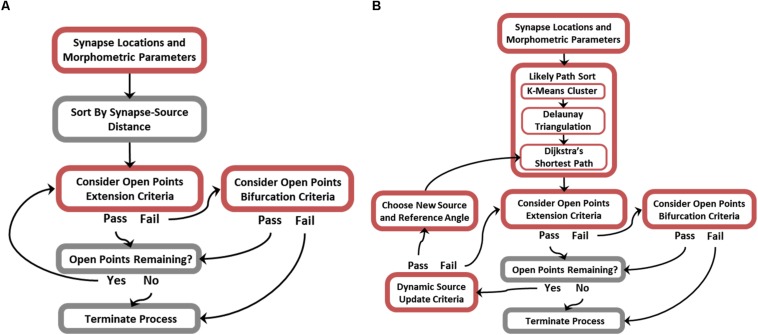
Flow diagrams describing the ROOTS algorithm. For greater efficiency, the generative algorithm was designed to operate in one of two modes: **(A)** Without or **(B)** with dynamic source-point updating criteria. This feature enables generated morphologies to successfully conform to acute turns of varying radius. Disabling this feature when not needed improves efficiency.

While many of the possible branching patterns of a functional arbor are constrained by the topography of synaptic targets, there remain as many as *n*^(*n*−2)^ trees that span a set of targets (*n*), few of which are biologically plausible (Cayley, 1889). It follows that encouraging biological realism requires additional non-trivial steps to constrain branching features of generated topologies. Figure 3 is an algorithm flow diagram and pseudocode (provided in Supplementary Figure S1) for proper preprocessing and successful execution of ROOTS. This process generated the trees analyzed in later sections of this manuscript. In brief, ROOTS seeks to minimize the quantity of membrane required to span a set of synaptic targets while satisfying user-specified branching criteria. These criteria (at the time of writing) include: branch extension angle (meander) and length (“Extension Criteria”), and bifurcation angle and length (“Bifurcation Criteria”). The method was also designed in a manner that allows additional global criteria (branch order, number of bifurcations, total length, etc.) to be designed and applied within ROOTS. The process by which this is accomplished involves serially considering sorted (by source–target distance or “Likely Path”; according to Figure 3) open points by alternating between branch extension (appending points to an existing branch) and bifurcation (beginning of a new branch). If in the process of extending a branch it is found that no points satisfy branch extension criteria, then the algorithm switches to bifurcation. If a bifurcation can be created according to bifurcation criteria, then the algorithm switches back to extending the newly begun branch. This iterative process continues until either extension and bifurcation criteria cannot be satisfied or no open points remain. Model inputs (synaptic targets, branching and bifurcating criteria, and global criterial) dually exert control over the emergent spatial/geometric features of the entire terminal field and the branching patterns that develop as the fiber is constructed.

While the core of this algorithm is capable of growing axons with highly particular geometries, it is limited in the cases where axons execute acute turns without forming connections that cross the resulting sulcus. An additional rule can be used to mitigate this case-specific flaw: a relative location sensitive dynamic source point and reference angle (Figure 3B). The principle difference between simplified and dynamic source updating modes (Figures 3A vs. 3B) is the way in which points are sorted and, therefore, the order in which points are considered for inclusion into the tree and the reference angles that are subsequently calculated. The “likely path sort” components in Figure 3B, effectively, allow fibers to be grown along manifold surfaces where the simplified algorithm is for efficient growth of conical or star-shape fibers. This approach requires additional preprocessing – replacing the initial sorting of points with respect to a single source point with a more sophisticated sort and a dictionary of relative source-points and reference directions. This new process is performed by (i) finding spatial clusters of synaptic targets using K-means, (ii) fitting a mesh to the spatial clusters using Delaunay triangulation, and (iii) discovering the most likely path to any cluster center from the origin source-point through the constructed mesh (Hartigan and Wong, 1979; Chew, 1989). The most likely path, found using Dijkstra’s shortest path algorithm, is then used as a lookup table where the edges leading to the cluster within which any new point being considered may be found are used to constrain angles of branch extension and bifurcation (Chen, 2003). Execution of these three new steps results in a path directed sorting of targets. It should be noted that K-means is the least critical new component and is only used to regularize the triangulation and reduce the complexity for the steps that follow (between 2500 and 3000 clusters were used in this implementation to have the desired effect). This new rule allows successful execution of acute turns, where the fiber bends backward toward the source point without crossing the resulting cleft or forming any cycles. Critically, these acute turns are executed without relaxing constraining parameters for branching and bifurcating.

While there are many clustering algorithms and many sophisticated meshing algorithms, K-means and Delaunay algorithms were selected because of their speed and reliability. Because dynamic sourcing (Figures 3B vs. 3A) adds considerable computational burden, it is valuable to select preprocessing methods which do not add to this burden needlessly. Supplementary Figures S2–S4 show development of this stage of the algorithm and its three constituents: K-means clustering, Delaunay triangulation, and Dijkstra’s shortest path methods.

### Synaptic Boutons

In recognition that axons have complex surfaces and are not just a series of smooth and simple pipes, additional functions were written to allow complexities such as synaptic boutons to be added to an already grown fiber. These boutons, as they are seen in the dentate perforant path at non-terminal presynaptic densities, are described by Tamamaki as “periodic varicosities” (Tamamaki and Nojyo, 1993). Each bouton is ≈5 μm long, ≈0.7 μm in diameter, with varying distances between, depending on the topography of the synaptic targets. Because neither the exact distribution of inter-bouton distances nor multisynapse formation behavior is known in this tissue system, it was assumed that they were uniformly distributed throughout the terminal axon arbors every 25 μm, with each bouton being 5 μm in length and 0.7 μm in diameter. Because these boutons are non-terminal they are likely to be actively conducting. Lacking experimental evidence to frustrate this assumption, sodium, calcium, and potassium channel densities and conductances were implemented with the same parameters in bouton compartments as in non-bouton axonal regions.

Following development and testing of this algorithm, fibers were exported for simulation in environments such as NEURON (Hines and Carnevale, 2003). Later in this paper, extracellular electrical stimulation studies performed with the generated morphologies are presented to demonstrate the maturity of this analysis pipeline.

### Arbor Simplification and Computational Complexity

Simulation of detailed neuronal models is computationally expensive. While this study was enabled by non-competitive access to a 4,040 processors computing cluster, there remain concerns about impractical and unnecessary model complexities. With as many as 17,700 possible synaptic connections made by each EC arbor, it became clear that an approach to morphology simplification would be necessary to reduce the computational burden of both generating and simulating arbors in NEURON. While the exact number is not known, because of the *en passant* nature of the perforant path fibers it is likely that a passing fiber synapses more than once with any target granule cell. This provides the opportunity to generate trees using fewer nodes due to the relative co-locality of synaptic connections between an arbor and any given granule cell. To examine this assumption, we generated arbors with 8,850 (two synapses per target cell) or 5,900 (three synapses per target cell) target cells rather than 17,700 target cells and used a single node from each to guide arbor growth. Perforant path fibers were generated using each of these node counts and then each was also line simplified. The Ramer–Douglas–Peucker (RDP) algorithm for line simplification was used on each of these trees to determine the minimum node count required to approximate full complexity fibers (Saalfeld, 1999). Using a fraction of the typical dentate granule cell dendritic arbor height and width (e.g., 20% or ∼45 μm) to set a maximum RDP-epsilon ensured that path deviations would be much less likely to make otherwise probable EC–DG connections anatomically impossible.

### Alternative Generative Models

To test the functionality of this algorithm in comparison with others that already exist, we attempted to create satisfactory arbors with two commonly used alternative tools which were originally designed to grow dendritic arbors: the TREES Matlab toolbox (Cuntz et al., 2010), and L-NEURON (Ascoli and Krichmar, 2000; Scorcioni and Ascoli, 2005).

The TREES toolbox, like ROOTS, depends upon carefully selected nodes or points to grow a graph; therefore, spanning trees generated by this method can use the same set of synaptic or cellular targets as those utilized by the proposed system. Unlike ROOTS, however, the only parameter that can be adjusted to improve the performance of the resulting morphology is the balancing factor (BF). This BF represents the weighting of priority for path length versus conductive delay in the spanning tree that is generated. To study this approach, a new topology grown via TREES utilizing the same control points as a ROOTS fiber. The BF was calibrated by minimizing a multi-objective function (MOF), formulated as an unweighted sum of independently normalized mean root square error for each of the following morphometrics: Euclidean distance/path-length (BF), branch order, bifurcation angle, and total path length. The BF of the arbor generated by TREES was set at the value that minimized the difference between the MOF scores of the arbors generated by each system. To maximize similarity between these two morphologies through calibration of the TREES BF, direct (Euclidean) vs. path length ratios were calculated for each carrier node, then a histogram of these data was fit with a kernel density estimate (KDE via Gaussian smoothing). This process was repeated for path length, branching order, branch length, and branching angle. The KDEs for direct vs. path length and branching angle for the TREES axon were subtracted from those for the ROOTS fiber. These differences were independently normalized and the RMSE was calculated. Each of these metrics were summed without any weighting. The TREES BF was then calibrated through minimizing the summed normalized root mean-squared error of these differences (*U*) according to Eq. 1.

(1) U=dBF+dBO+dPL+dBL+dBA

where each term represents the normalized summed difference of BF (dBF), branching order (dBO), total path length (dPL), branch length (dBL), and bifurcation angle (dBA). Unlike TREES and the proposed algorithm, L-NEURON doesn’t rely on a preselection of target nodes to construct a topology, but rather depends upon measures of branching structure of a tree, or morphometrics. Fitted distributions to these measurements are then stochastically sampled to grow a tree. This approach is intended to capture branching patterns without much regard for the emergent spatial features of a tree. Because of the lack of an extensive database of EC axons from which to take branching measurements, we resorted to using the companion tool L-MEASURE to extract morphometrics from one of our own generated arbors to gauge the feasibility of using stochastic methods to generate axonal arbors when such a dataset does become available (Scorcioni et al., 2008). Extracted morphometrics were then fed to L-NEURON to generate a morphometrically equivalent arbor.

Virtual arbors were ultimately evaluated based on their ability to capture known spatial features of EC axon terminal fields, including a complex geometry which conforms to the contours of the dentate gyrus.

### Strength–Duration Relationship in Response to Extracellular Electrical Stimuli

To understand how fiber geometry in the hippocampus gives rise to patterns of activity, fibers with varying patterns of diameter were simulated in response to a range of current source–arbor distances and stimulus amplitudes. Images of spiny stellate fibers from Tamamaki and Nojyo (1993) and more evidence from Bindocci et al. (2017) show continuous fibers with “periodic varicosities,” or non-terminal synaptic boutons on the *en passant* fibers. Because fiber diameter has been shown to influence both conduction velocity and excitability, it was important to explore the response characteristics of fibers with this sub-micron variation in diameter (Clark and Plonsey, 1970). A fiber was generated by ROOTS and instantiated with one of three patterns of diameter in the simulation environment, NEURON 7.6.2, so that its behavior could be simulated (Hines and Carnevale, 2003). Hodgkin–Huxley membrane biophysics under *in vivo* temperature conditions were inserted in all compartments and d-lambda rules were used to determine appropriate space constants for compartmentalization of the fibers (Hines and Carnevale, 2003). All other biophysical features were borrowed from nodal biophysics described in Johnson and McIntyre (2008). An itemized table of biophysical properties can be found in Supplementary Figure S5. All morphological features for these fibers, other than the deliberate variations in diameter, remained as presented in Table 1.

Monopolar point-source stimuli were used in all stimulation experiments presented in this article. Electrodes were placed in one of two locations near the primary bifurcation of the perforant path at distances of 100 and 500 μm from the nearest neuronal compartment.

Following model construction, two sets of analysis were performed. The first comprised a strength–duration study of complex arbors. Square-wave pulses of anodic or cathodic charge polarities (+ and −, respectively) with widths between 0.025 and 1.4 ms were delivered at each of the two distances. The extracellular voltage throughout the model space was estimated using an analog of Coulomb’s law with material resistivity of 3.8 Ω-m (Eqs 2 and 3) (Clark and Plonsey, 1970; Holt and Koch, 1999; Bingham et al., 2018).

(2) $$\phi \,\left(x,y,z\right)=\frac{I_{0}}{4\,\pi^{*}\,\sigma_{i}^{*}\,r_{i}}$$

where Φ is the field potential resulting from a current source, *I*. The conductance, σ, is the inverse of resistivity. Radial distance, *r*, is found by Eq. 3:

(3) $$r_{i}=\sqrt{{\left(x_{i}-x_{0}\right)}^{2}+{\left(y_{i}-y_{0}\right)}^{2}+{\left(z_{i}-z_{0}\right)}^{2}}$$

By adding new voltage sources in series with the circuital elements representing each section of membrane, extracellular potentials calculated via Eqs 2 and 3 were applied to neuronal compartments within the NEURON model using the “extracellular” mechanism. Stimulation was delivered over a range of current amplitudes designed to cover, at a minimum, rheobase to twice rheobase for each set of stimulating conditions.

The second analysis examined the same models and stimulating setup but focused on the temporal distribution of the response at rheobase. The time it took each compartment in the fiber to reach action potentiation was recorded, used to populate a histogram, and then Gaussian smoothed to present a KDE. These plots were used to identify the impact of boutons on conduction velocity throughout the complex arbor.

### Comparison With Implied Axon Conduction Latency Estimates

In order to demonstrate the differences between the relatively sophisticated axonal models grown via ROOTS and more simple axon representations, a simple distance-based delay was estimated based on the average conductance velocities of the ROOTS fibers and visualized using KDEs after the manner previously described. The distance-based conduction delay mechanism just explained is described in figures and relevant results sections as a “pure-delay” mechanism because no explicit cable model is used to approximate the delay. To clearly demonstrate the differences that are not readily seen in the KDE, pair-wise differences (residuals) of compartmental APs using ROOTS and the pure-delay mechanism were measured and plotted.

### Data/Model Sharing

It is important to us that the method by which functional cortical axons were generated be highly accessible to other members of the computational neuroscience community, especially those studying neuromodulation for the treatment of diverse neurological disorders. Therefore, the graph-based algorithm used in this study has been compiled in a user-friendly manner and distributed to the Python Package Index under the name *Neural Roots* (*Roots*)^1^ ^,2^ along with concise documentation. Neural Roots was written with Python 3.6.9 and has a few non-standard dependencies, including: SciPy, NetworkX (Hagberg et al., 2005), Mayavi (Ramachandran, 2001), Shapely, and Pandas (McKinney, 2010) (searchable in PyPi, the Python Package Index). While some backward compatibility is likely, this has not been extensively tested. The model is also being prepared for submission to ModelDB where it will be available alongside other elements presented in this paper.

## Results

Results comprise three parts: (i) a qualitative and quantitative assessment of the proposed algorithm, (ii) a comparative analysis with alternative methods, and (iii) the presentation of strength–duration curves and an analysis of EC fiber recruitment order and temporal distribution of the response to extracellular stimulation.

### Algorithm Assessment and Validation

**FIGURE 4:**
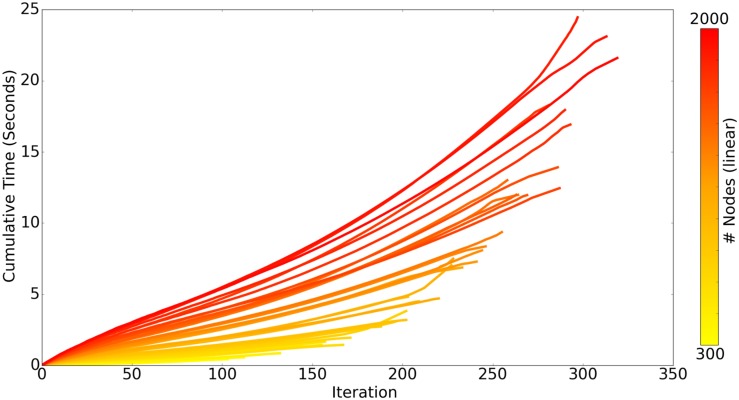
Each line represents the time-course of ROOTS execution to generate an axonal graph with dentate perforant path topography using different numbers of carrier-points, ranging from 300 to 2000. Each iteration of the algorithm includes one loop for branch extension and another for bifurcation. While the above plot presents performance while growing a specific type of arbor, method performance is highly dependent upon input parameters and may be faster or slower for other model types.

There are two chief loops within the core ROOTS algorithm: branch extension and bifurcation. As the algorithm proceeds, the complexity of branch extension decreases while that of bifurcation increases, this can be seen in Figure 4 as a linear/slightly supra-linear shape of each individual line. Bifurcation criteria become more difficult to satisfy as the axon graph becomes more complete, resulting in exponential increases in time-to-completion when larger and larger axons (more nodes to connect) are generated. Many aspects of these trends are dependent upon the spatial topography of the nodes themselves. For example, if nodes fall along a straight enough line, a single branch extension loop with no bifurcations will complete the axon graph and the time-to-completion will be perfectly linear with respect to the number of nodes.

### Arbor Simplification and Computational Complexity

Consideration of the complexity of the resulting fiber is essential because simulation of just a few of these fibers at full complexity could be highly taxing on a single-processor computer. Supplementary Figure S6 demonstrates the relationship between simulation efficiency and fiber complexity using the NEURON engine. Simulation efficiency is reported as the ratio of clock-time to simulated time. As fiber complexity increases, the amount of processing time (clock-time) required to complete an otherwise controlled simulation also increases linearly. Data for Supplementary Figure S6 were collected on an Acer Aspire TC-885-UR17 desktop computer.

**FIGURE 5:**
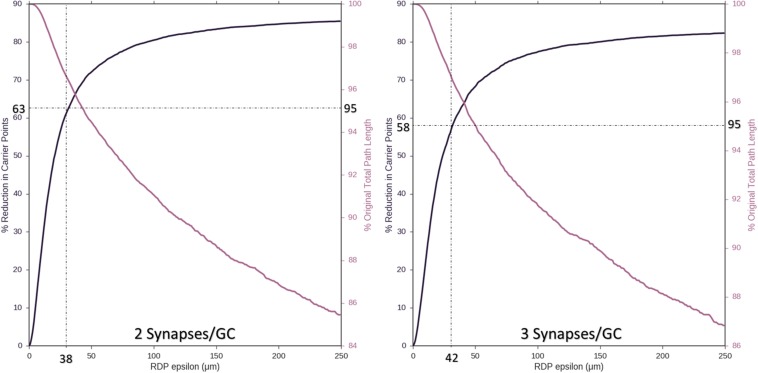
As an approach to reducing computational complexity, Ramer–Douglas–Peucker (RDP) line simplification was studied over a range of epsilon (the maximum distance a simplified curve can be removed from an the original), for reduction in node count (**Left** *y*-axis) and change in total path length (**Right** *y*-axis). Simplification was performed for the cases where two or three synapses were made with each targeted cell (8,850 or 5,900 starting nodes, left and right plots). Above 95% path length is preserved by setting epsilon to 38 and 42 μm, resulting in reduction of required number of nodes to 2,944 and 2,097, respectively (60%).

Ramer–Douglas–Peucker line simplification was performed on axons with two or three synapses per target granule cell (8,850 or 5,900 nodes). Figure 5 demonstrates that axons with as few as 2,944 and 2,097 target nodes can be used to approximate the behavior of an arbor with maximum complexity, 8,850 and 5,900 target granule cells, respectively. These simplifications can be made while allowing, at most, 38–42 μm deviations from original contours and <5% reduction in total path length.

**FIGURE 6:**
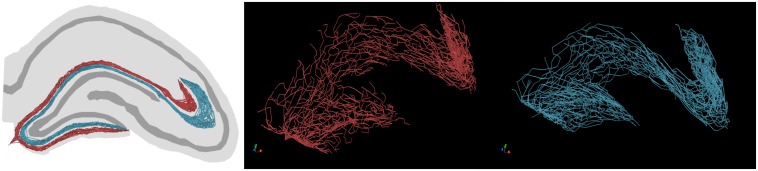
Example graph models (red, LEC; teal, MEC) of entorhinal cortical axon terminal fields, generated by the proposed algorithm. These axons have features expected in *in situ* fibers, namely: *en passant* terminal topography, laminar architecture (i.e., MEC/LEC are spatially segregated within the dentate molecular layer), and fibers proceed to the ends of each supra/infrapyramidal blade of the dentate gyrus and proceed to a terminal field in CA3/2/1.

Example graph models of EC axon terminal fields which incorporate these simplifications generated by the proposed algorithm are presented in Figure 6. The algorithm described in Figure 3B yielded fibers that captured the known features of layer 2/3 EC spiny stellate axons which make up the dentate perforant path (Table 1). These features include but were not limited to: distribution of bifurcation angles of approximately 80±34°; a septal–temporal range of between 1 and 1.5 mm; laminar organization with the MEC and LEC in the outer and middle thirds of the dentate molecular layers, respectively; saturation of branching order at a reasonable level to encourage *en passant* synapsing; presentation of both DG and CA3 terminal fields with the preservation of a fissure between the two fields; and finally, the tree structure passed through a plausible topography of DG and CA3 arbor domains which enables *in situ* levels of connectivity.

### Alternative Generative Models

A morphology generated via our own graph-based algorithm (Figure 3B) was selected and morphometrically described via L-Measure and then, using the stochastic system called L-Neuron, attempted to regenerate a morphometric equivalent. This exercise failed to return a morphology which could conform to the topography of the molecular layer of the dentate gyrus. The chief reason being that purely stochastic methods are unable to prevent excursion of fibers beyond natural boundaries in the volume of tissue system being modeled. The general stochastic method is, therefore, unsuitable for generation of anatomically appropriate axonal morphologies without further modification to constrain emergent spatial characteristics.

**FIGURE 7:**
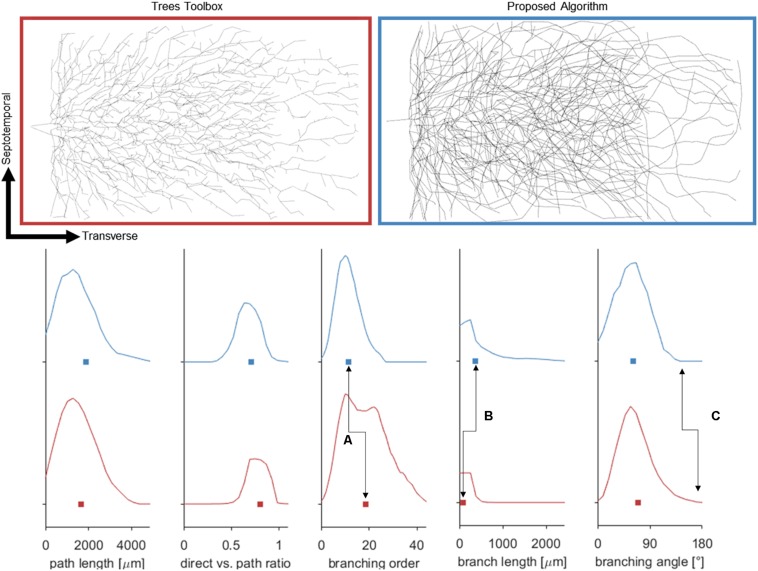
**(Top)** Dorsal aspect of the enclosed blade (suprapyramidal) portion of an entorhinal cortical axon generated by one of two methods: Trees Matlab toolbox (BF = 5), and the proposed algorithm. Balancing factor, and connection threshold for the Trees toolbox were selected to align these distributions aligned favorably. **(Bottom)** Fundamental statistical distributions comparing the two methods in terms of nodal path length, direct distance vs. path distance, branching order, branch lengths, and bifurcation angles. The plotted curves represent probability densities and the box represents the mean. Despite matching many features of the proposed algorithm, the Trees generated arbor does not capture the *en passant* nature of the perforant path [seen in branching order (A), and length (B)] and has lingering bifurcations which have extreme angles (C).

**FIGURE 8:**
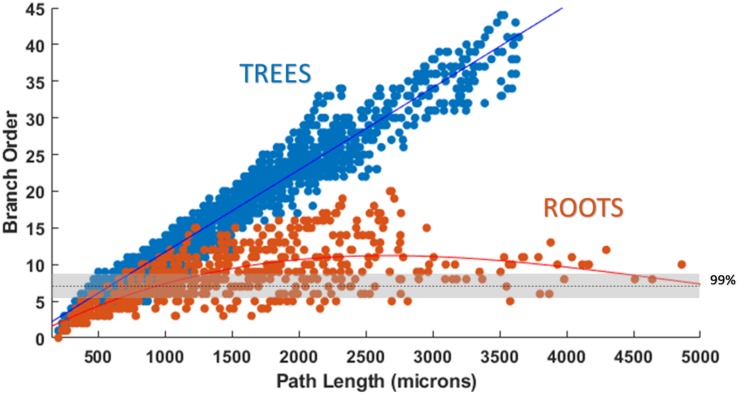
Plotting path length vs. branch order makes it clear that ROOTS more accurately captures the *en passant* nature of the perforant path than does TREES. Each dot represents the branch order of a NEURON section. 99% of total axon path length of entorhinal cortical spiny stellate cells should be achieved with fiber of branch order no greater than ≈7 (Budd et al., 2010). Increased levels of early terminations in TREES is a geometric challenge that may have significant implications for extracellular stimulation (Joucla and Yvert, 2009).

Attempts were also made to generate accurate morphologies using the TREES MatLab toolbox. These efforts yielded trees superior to those generated by the L-Neuron method, though still deficient in important ways. An axon generated by TREES was selected for quantitative and qualitative comparison with that yielded by earlier analysis using the ROOTS system (Arbor Simplification and Computational Complexity). Each tree was constructed using the same set of target nodes; therefore, all differences in patterns of branching arise from differences between the two algorithms. The TREES BF was then calibrated through minimizing a MOF, attempting to find the best possible match between TREES and ROOTS arbors. This process yielded a functional axon from the TREES algorithm, though differences remained that could not be resolved through manipulation of the BF alone (Figure 7). Despite a high degree of similarity in direct (Euclidean) vs. path length, total path length, and branching angle distributions, TREES resulted in a higher than expected number of terminal branchlets. This shifted the branching order and branch length distributions to the right and left, respectively. These shifts demonstrate difficulty for the TREES approach in appropriately capturing the *en passant* connection schema which typifies EC axons of the perforant path, where terminal branchlets are reportedly rare. Figure 8 (branch length vs. branch order) further highlights this conformational difference between the two fibers and provides a useful comparison to experimental measurements. In this figure, the black line denotes the branch order at which 99% of the total path length was achieved in layer two spiny stellate axons reported by Budd et al. (2010). While the comparison with Budd’s report is useful, it is made with hesitancy because of the variability of branching that occurs within the long stretch of fiber between the entorhinal cortex and the extensively branching terminal arbor. The difficulty TREES has in controlling branch order arises from the paradigm of control implemented in the system. More specifically, the balancing of total path length versus conduction time (BF) does not provide for fine control of the shape and branching properties of an arbor within a volume. The significance of this limitation of TREES with respect to ROOTS is particularly clear when considering the implications for extracellular stimulation where it is thought that terminations and thorough-fare fibers behave differently in the presence of extracellular electric fields (Joucla and Yvert, 2009).

**FIGURE 9:**
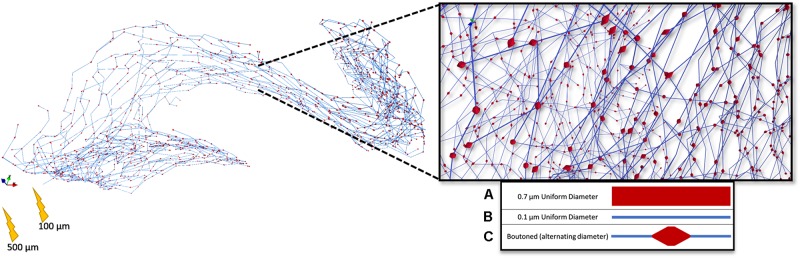
Rendering of a highly branched perforant path axon with presynaptic boutons dispersed evenly throughout the terminal field. 5 μm boutons were spaced 25 μm apart according to Tamamaki and Nojyo (1993). This arbor, with three different representations (A–C) of diameter, was then used in a set of stimulation experiments to study the impact of microstructure geometry on emergent patterns of spatiotemporal patterns of activity.

An additional challenge not satisfied by the TREES approach is that of performing acute turns. A portion of the perforant path innervates the CA3/CA2 region of the hippocampus and therefore must be able to extend from the end of the enclosed blade of the dentate into these other domains. The algorithm presented in Figure 3B (dynamic source) describes how updating reference angles allow the fiber to bend around complex anatomies successfully without violating morphometric constraints (Figures 6, 9). An attempt to replicate this feat using the TREES Matlab toolbox is presented in Supplementary Figure S7.

### Strength–Duration Relationship in Response to Extracellular Electrical Stimuli

**FIGURE 10:**
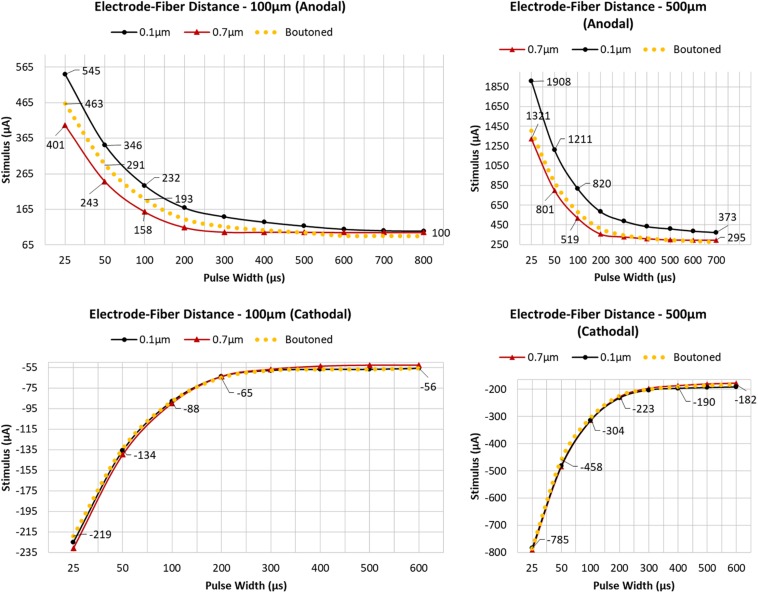
Arbors of three patterns of diameter corresponding to stem and bouton diameters observed by Tamamaki and Nojyo (1993), and a fiber with appropriately distributed boutons, were simulated in response to cathodal pulses (25 μs–1.25 ms) and a range of stimulus amplitudes from distances of 100 and 500 μm **(Left, Right)** Tamamaki and Nojyo (1993). Larger fibers have shorter chronaxies than small and this difference is exaggerated by large electrode–fiber distances and longer pulse-widths. 0.7, 0.1, and Boutoned legend keys correspond to A, B, and C in the Figure 9 legend.

To determine the importance of including boutons when simulating extracellular electrical stimulation of axon models, strength–duration curves for three diameter patterns of a ROOTS arbor (generated via the algorithm described in Figure 3B), where diameters correspond to bouton and inter-bouton sizes (according to Figure 9), were estimated and presented in Figure 10. When stimulating with anodal pulses, these results agreed with previous reports that larger diameter fibers have shorter chronaxies than small, and that this difference is exaggerated by large electrode-fiber distances and longer stimulation pulses. For cathodal impulses of such small and highly branched fibers, the model yielded negligible opportunity for selective activation of fibers by diameter. This protocol was repeated for a biologically realistic arbor, complete with boutons. The recruitment pattern of the boutoned fiber was no different in a cathodal field but under anodal conditions had activation thresholds between those of the 0.7 and 0.1 μm uniform diameter arbors. The differences in threshold between the boutoned fiber and the 0.7 μm fiber decreased with increasing electrode–fiber distance. These differences dissolved as the pulse-width approached 700 μs.

**FIGURE 11:**
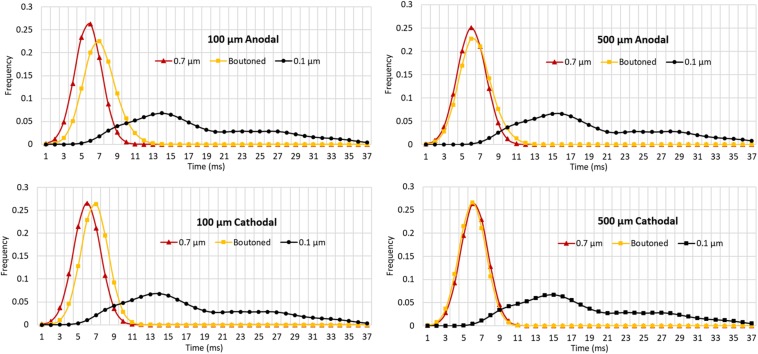
Kernel density estimates (1.5 ms Gaussian kernel) for compartmental action potential delays in perforant path arbors with a diameter of 0.7 and 0.1 μm, and a boutoned fiber when stimulated at rheobase amplitude for 1 ms. From **top** to **bottom**, **left** to **right**, showing two electrode-fiber distances and anodal and cathodal stimulation. Excepting at very close distances, boutoned fibers had temporal activation features best approximated by a fiber with uniform 0.7 μm diameter. 0.7, 0.1, and Boutoned legend keys correspond to A, B, and C in the Figure 9 legend.

The impact of boutons on temporal dynamics are presented in Figure 11. The time of first action potential is plotted along the horizontal axis for each neuronal compartment in the biologically realistic arbor with the vertical axis representing probability density (frequency). A fiber with each of three patterns of fiber diameters (uniform 0.1, 0.7, or boutoned) were simulated in response to 1 ms stimuli at rheobase amplitude. Activity was initiated near the electrode and then actively conducted to other compartments, including those beyond the effective volume of the extracellular electric field where artifact voltage dropped to tens of microvolts. In Figure 11 are plotted, as a KDE, the time-course of threshold activity of all compartments in each case. As the average fiber diameter is increased, the shape of the KDE shifts to the left, indicating that activity in the arbor is occurring with less delay following stimulation. This shift is explained by the increased conduction velocity throughout the arbor which results from changing compartment diameters. Average conduction velocities were 0.88, 0.53, and 0.12 m/s in the 0.7 μm, boutoned, and 0.1 μm fibers, respectively. The boutoned fiber nearly mirrored temporal patterns of activity of the uniform 0.7 μm fiber, except at very close electrode–fiber distances.

### Comparison With Implied Axon Conduction Latency Estimates

**FIGURE 12:**
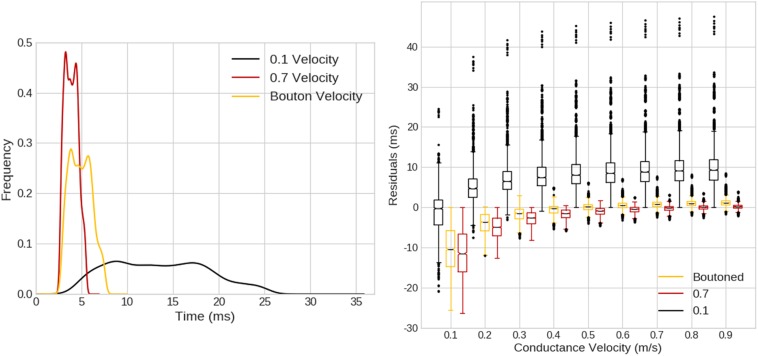
**(Left)** Kernel density estimates (1.5 ms Gaussian kernel) for expected compartmental action potential delays calculated based on the Euclidean distance from the site of action potential initiation (obtained from ROOTS fibers simulated in Figures 10, 11) to compartment centroids. Three cases are visualized based on the average conductance velocity calculated from fiber behavior in the ROOTS explicit fiber at threshold (100 μm-case). Average velocities were 0.88, 0.53, and 0.12 m/s, for 0.7 μm, boutoned, and 0.1 μm ROOTS fibers, respectively. **(Right)** Pair-wise compartmental latencies were compared between a distance-based delay mechanism and explicit fiber representation via ROOTS across a range of conduction velocities plotted along the *x*-axis. As the conduction velocity moved nearer to the average velocity of the ROOTS fiber, average residuals decreased. Error was largest when comparing the smallest ROOTS fiber, but differences were still on the order of milliseconds near ideal conduction velocity for the faster conducting ROOTS fibers.

When compared with a pure-delay mechanism, as in Figure 12 left, ROOTS fibers exhibited a smoother distribution of compartmental Aps, though the KDEs for the pure-delay mechanism had similar shape and length. When exploring the impact of varying the conductance velocity on pair-wise compartmental AP latencies between the pure-delay mechanism and each of the ROOTS fiber geometries, the role of fiber topology on the precise latencies of conduction to each compartment is more obvious. Especially when comparing the pure-delay mechanism estimate to the 0.1 μm ROOTS fiber behavior (Figure 12 right), very large differences in AP latencies emerge. This demonstrates how sensitive this test is to conduction velocity alone but also illustrates the importance of accurate representations of fiber microstructure features and arbor topology. Even as the differences are reduced in comparisons with larger and faster conducting fibers, errors of several milliseconds remain. Even should a modeler be so lucky to correctly select a proper conduction velocity (not to mention correctly predicting the site of action potential initiation), differences are difficult to eliminate without more sophisticated methods of approximating the path length to each compartment.

## Discussion

Together with increased computing power, more robust repositories of electrophysiological and histological information have created opportunities for neural models of greater complexity. Despite these ever-improving sources of data, biologically realistic neural modeling has outpaced experimental studies in many areas of inquiry, providing a testbed for new hypotheses as well as a wealth of preliminary data to aid the design of superior *in vitro* or *in vivo* studies. However, the mismatch in biological realism between experimental and computational modeling, and continuing ambitions to advance computational neuronal modeling, creates a need for sound approaches to generate functional neuronal models that can be refined as the quantity and quality of experimental data improve. The modeling approach presented in this paper demonstrates this utility.

### General Application of ROOTS

This manuscript has demonstrated the application of ROOTS to a single fiber system, but this process could be followed for the generation of appropriate models from other anatomical regions with different morphological features. This is true because the criteria used by ROOTS are generally applicable to axon terminal arbors that form a spatial network by connecting parent cells to synaptic targets with a unique branching pattern. The degree of accuracy that can be obtained with ROOTS is directly linked to the quality of anatomical data available to constrain the method. There are three basic tasks that must be accomplished to generate usable models: identify the topography of synaptic targets, identify the branching properties of terminal arbors, and identify the volume from which arbors begin. While these tasks present a challenge, none are as difficult as describing and extracting enough explicit reconstructions from histology to fully recapitulate a large-scale pathway model.

Identifying the topology of synaptic targets, at its most basic, means combining knowledge of the spatial range of terminal arbors with an estimate of the number of terminal boutons expected for each fiber. Identifying branching properties of terminal arbors requires reduction of experimental observations of these terminal arbors to a statistical distribution of bifurcation angles, at the very least. Knowledge of the number of bifurcations, total fiber length, meander angles, and inter-bifurcation lengths is also useful. Lastly, identifying the volume from which arbors initially bifurcate is important in order to force a fundamental directionality on generated arbors. Each of these steps yields important parameters that ROOTS expects and uses to encourage realism in the morphologies that are output by the software. Should one of these specific data not be available, the modeler should not be discouraged from attempting to use ROOTS anyway, as each statistic is merely one part of the puzzle ROOTS seeks to solve. Generated fibers can still be a useful stand-in and can always be augmented as superior data become available.

### Limitations and Alternative Methods

Despite the demonstrated functionality of the model presented herein, it is not without limitations. First among potential limitations is biological realism, which may still be limited due to lack of accurate and well described experimental measurements of fibers from hippocampal tissue samples. The clearest limitation (though simultaneously the chief motivation for development) of this modeling approach and the study presented in this paper is the lack of an extensive dataset describing EC axons in the perforant path, including rich morphometrics. Existing explicit reconstructions have readily identifiable errors and, therefore, should not be used as solitary sources of branching morphometrics or be virtualized and used in stimulation models without sophisticated automated and/or manual repair (Tamamaki and Nojyo, 1993; Budd et al., 2010). The issues include slicing artifacts which distorts both distances along or across serial slices and bifurcation angles where branches span multiple slices; angle and distance mismeasurements due to imaging along a single axis and, therefore, failing to correct for the impact of rotation of neural processes with respect to observer perspective; and failures of automated algorithms which may result in orphaned sections or even cycles in the final tree (Quilichini et al., 2010). Despite the unavailability or verifiability of these data, the modeling methodology presented in this study represents the most detailed and sophisticated functional model of layer 2/3 EC spiny stellate dentate perforant path axons to date. In general, the utility of this modeling approach is most apparent for cases in which explicitly reconstructed morphologies are sparse, poorly described, and the fibers to be modeled have complex geometry and branching structure. Further, the parameterization of the model is such that, should this data later become available, the generative morphologies could be updated to reflect new knowledge of *in situ* morphometrics.

While the present study espouses a ROOTS, there are many algorithms that might achieve topological characteristics reminiscent of neuronal branching.

Although existing stochastic models may be effective for dendritic arbor generation, they are not naturally adapted to the construction of axonal morphologies because they fail to reliably conform to pre-determined geometries or volumes that are irregular or highly non-symmetrical (Rozenberg and Salomaa, 1980). The ability to conform to a predetermined volume or geometry is a particularly important feature for axons in the hippocampus, which often have lamellar organization between layers and laminar organization within their terminal fields. For stochastic models to accomplish this, new pre-processing intensive and potentially inefficient volumetric constraints would be required, adding to algorithmic complexity (e.g., randomly walking line that must also connect arbitrary points).

When guided by serial histological sections, spanning-tree algorithms provide an efficient approach to reconstructing spatial trees. However, without some modification, traditional minimum-spanning-tree algorithms can result in spatial error and distorted branching patterns (Budd et al., 2010). Using higher resolution images forces significant biological realism onto the resulting graph, making error more manageable. However, minimum-spanning-trees generated from points sampled at random from a volume representing an axon terminal field are less biologically realistic due to the extreme path lengths that result. To moderate this outcome, some investigators have proposed a spanning-tree algorithm which balances the minimization of membrane against path length (Cuntz et al., 2007, 2010; Budd et al., 2010; Budd and Kisvarday, 2012). However, optimizing this balance is known to be an np-hard problem due to the direct conflict introduced by including these two variables in the same loss function (Hu, 1974; Alpert et al., 1995; Khuller et al., 1995; Wu et al., 2000; Gastner and Newman, 2006). Another challenge to using path-length/membrane-minimization balancing algorithms is that it only allows indirect control over important tree morphometrics such as bifurcation angle, branch extension angle, and inter-bifurcation length. Prominent implementations of this approach do not allow explicit morphometric thresholds to be set and, therefore, extreme branching patterns remain possible. Inflexibility and lack of sufficient parameterization represent significant limitations of these prior efforts because branching patterns directly impact spatiotemporal patterns of activity.

It is plausible that established stochastic methods, known to be highly proficient at recapitulating tree morphometrics, could be modified to allow directedness and improved conformation to terminal field volumes. Despite these feasible and time-worthy alternatives, the model presented in this study has demonstrated sufficient performance in terms of accuracy, flexibility, and computational complexity.

While many alternatives to RDP for tree simplification exist, this method was preferable to other node cluster-and-merge methods (e.g, Kruskal’s algorithm) because these alternatives have the potential to introduce large differences in branching patterns, creating additional challenges in evaluating the equivalence of simplified and unsimplified fibers. Evaluation of the RDP simplification approach could be done in a very straightforward fashion because line simplification doesn’t result in changes in branching patterns—simplified arbors were compared to the reference case on the basis of path length and subjective evaluation of an acceptable RDP-epsilon, or the maximum distance between original and simplified contours.

Analysis in this paper involving extracellular field estimations used analytical methods that fail to completely account for anisotropy and heterogeneous resistivity of the tissue volume or full-wave propagation of electric fields. More sophisticated field estimation techniques (e.g., finite element, finite volume, boundary element, or admittance methods) that account for complex impedance or by filtering point-source estimations to account for amplitude dampening and phase-shift of stimuli could have been used but were deemed unnecessarily complex for the principle questions under examination in this article (Bossetti et al., 2007; Al-Humaidi, 2011). It should be noted, however, that rheobase predictions result from very long stimulus pulses, where >95% of the frequency domain signal falls <1 kHz; it follows that tissue capacitance is only a marginal source of error in these estimates of EC axon chronaxie. With respect to resistivity, this exercise is further justified because resistivity measurements in the region performed by López-Aguado et al. (2001) show nearly uniform resistivity throughout the molecular layer of the hippocampus; further, because the current source in these experiments was placed relatively far from resistive boundaries, current shunting distortions should be minimized in our estimations of electric fields in the volume occupied by EC fibers (Bingham et al., 2018). While future work not limited by these assumptions will be performed, doing so here falls outside the scope of the present study.

The study of strength versus duration of stimulus in the measurement of activation thresholds as performed in this paper does not provide conclusive data which concretely establishes the connection between fiber size and excitability for hippocampal networks. This is due to the realistic elements still missing from the models used, including non-uniform diameters of fibers, irregular bouton geometry and volumes, varying bouton topography, and an electrophysiological study of any differences in channel density and dynamics between boutoned and non-boutoned axon regions. Despite these limitations, it seems important to consider what impact boutons may have on fiber excitability within the terminal fields of small, highly branched, and unmyelinated axon fibers. Importantly, these results imply that action potentials in extracellular anodally stimulated axon terminal fields are initiated in boutons with lower input resistance than nearby portions of the fiber and that failing to consider the impact of boutons on conduction velocity will likely result in meaningful temporal errors. However, thorough experimental work is needed to confirm this possibility. It is further valuable to recognize that for long pulses delivered at more distant locations uniformly large diameter fibers approximate the vastly more complex fully boutoned fibers quite well and may provide a viable approach to reducing the computational demands of models that include detailed axon terminal arbors.

While much remains to be done, this study represents a step forward for detailed computational modeling of complex neuronal systems. Where previous models were either focused on peripheral axons with less complex arbors or used sophisticated methods to generate dendritic trees but neglected axons altogether, the model presented here demonstrates an approach to constructing functional axonal morphologies that can be used for diverse applications, including extracellular electrical stimulation of the cortex. It should be noted that while ROOTS is itself general, expert knowledge of the tissue system being virtually recapitulated is required: branching criteria and synaptic or cellular targets must be provided to the tool. Critical morphometric parameters include bifurcation angles, branch extension angle, and internode length; these must be independently determined by users. Despite these requirements, ROOTS presents many opportunities to increase the sophistication of model-based studies of neural tissue system dynamics and central nervous system neuromodulating devices such as deep brain stimulation or potential hippocampal memory prostheses (McIntyre, 2009; Hampson et al., 2018). ROOTS could also be used to support models of optical or pharmaceutical neuromodulation (Foutz et al., 2012; Bouteiller and Berger, 2017). The model framework could easily be extended to incorporate high resolution imaging data or combined with more complex volume conduction (or diffusion, diffraction, etc.) models to study tissue–electrode interactions at smaller spatial scales (Lujan et al., 2013). In addition to supporting prosthesis design, ROOTS facilitates model-based exploration of the effect of diseases which remodel axons on proper function of hippocampal tissue (e.g., multiple sclerosis) (Michailidou et al., 2015). More generally, ROOTS supports efforts to create networks of neuronal models for the study of biologically plausible spatiotemporal patterns of activity.

## Author Summary

As computer technology matures, constructing virtual models of brain parts has become an increasingly valuable approach to understanding how patterns of activity emerge in different neuronal structures. Many efforts to model populations of neurons have emphasized the implementation of biological realism for cell bodies and dendrites while settling for simplistic representations of axons. This neglect leads to potentially large errors in predictions of when and where synaptically driven activity in a neural circuit might occur. To address this concern, we have developed a novel algorithm called ROOTS to generate biologically realistic axon models for use in computer simulations of the brain. With realistic axons in place, such models can be used to predict how different regions of the brain respond to stimulation from implanted electrodes as part of a prosthetic device. This improvement in the realism of tissue models of the brain will provide superior support to on-going work to reveal the mechanisms of brain disorders and to optimize devices or drugs that are used to treat them.

## Data Availability Statement

All datasets generated for this study are included in the article/Supplementary Material.

## Author Contributions

CB designed the models and algorithms, conducted the analysis, and prepared the manuscript. AM and J-MB provided support for the design, analysis, and manuscript preparation. J-MB, DS, GL, and TB oversaw the project scope, project conception, and worked to maintain funding to support the project.

## Conflict of Interest

The authors declare that the research was conducted in the absence of any commercial or financial relationships that could be construed as a potential conflict of interest.

## Footnotes

1 https://github.com/bingsome/roots

2 https://pypi.org/project/Roots/

## Supplementary Material

FIGURE S1 Pseudocode of the core algorithm as presented in this study. Other components such as source point reassignment, and topology simplification procedures are executed in series with (before or after) the steps outlined here.

FIGURE S2 Clustering is performed via K-means clustering method. This regularizes the spatial properties of the mesh which will comprise the volume of the arbor and reduces computational complexity of subsequent steps.

FIGURE S3 Delaunay triangulation to construct a surface from spatially clustered synaptic targets. This yields a network from which ideal paths to potential target zones can be calculated.

FIGURE S4 Plotting of each “most likely path” between nodes in the Delaunay triangulated network of k-means cluster centers. Paths are constructed via Dijkstra’s algorithm. These paths are provided as inputs to the ROOTS algorithm to loosely guide branching behavior.

FIGURE S5 A brief table of biophysical parameters used in simulation of NEURON models. The only explicitly varying parameter (those not differing due only to changing compartment areas) was extracellular axial resistance. This followed the pattern implemented in Johnson and McIntyre (2008) to ensure this value was sensitive to fiber diameter.

FIGURE S6 Entorhinal cortical axons may form as many as 17,700 synapses with granule cells. However, growing axons with this number of nodes is slow and simulating axons with this complexity is computationally prohibitive when attempting to simulate *in situ* scale/density tissue models.

FIGURE S7 Example graph models (red, LEC; teal, MEC) of entorhinal cortical axon terminal fields, generated by the TREES toolbox method. These axons were generated with the addition of a terminal field in CA3/2/1. For very deep folds with small clefts, the TREES MST method has difficulty suppressing trans-cleft connections that short the cortical circuit without inappropriate manipulation of the number and density of targets, further exacerbating the proliferation of terminal branchlets.

VIDEO S1 This video shows the spatiotemporal pattern of activity following stimulation of an axon terminal arbor generated with ROOTS.
