# Supplementary figures and images for: ROOTS: An Algorithm to Generate Biologically Realistic Cortical Axons and an Application to Electroceutical Modeling
Source: Front Comput Neurosci. 2020 Feb 21;14:13. doi: 10.3389/fncom.2020.00013 (PMC7047217; doi:10.3389/fncom.2020.00013)

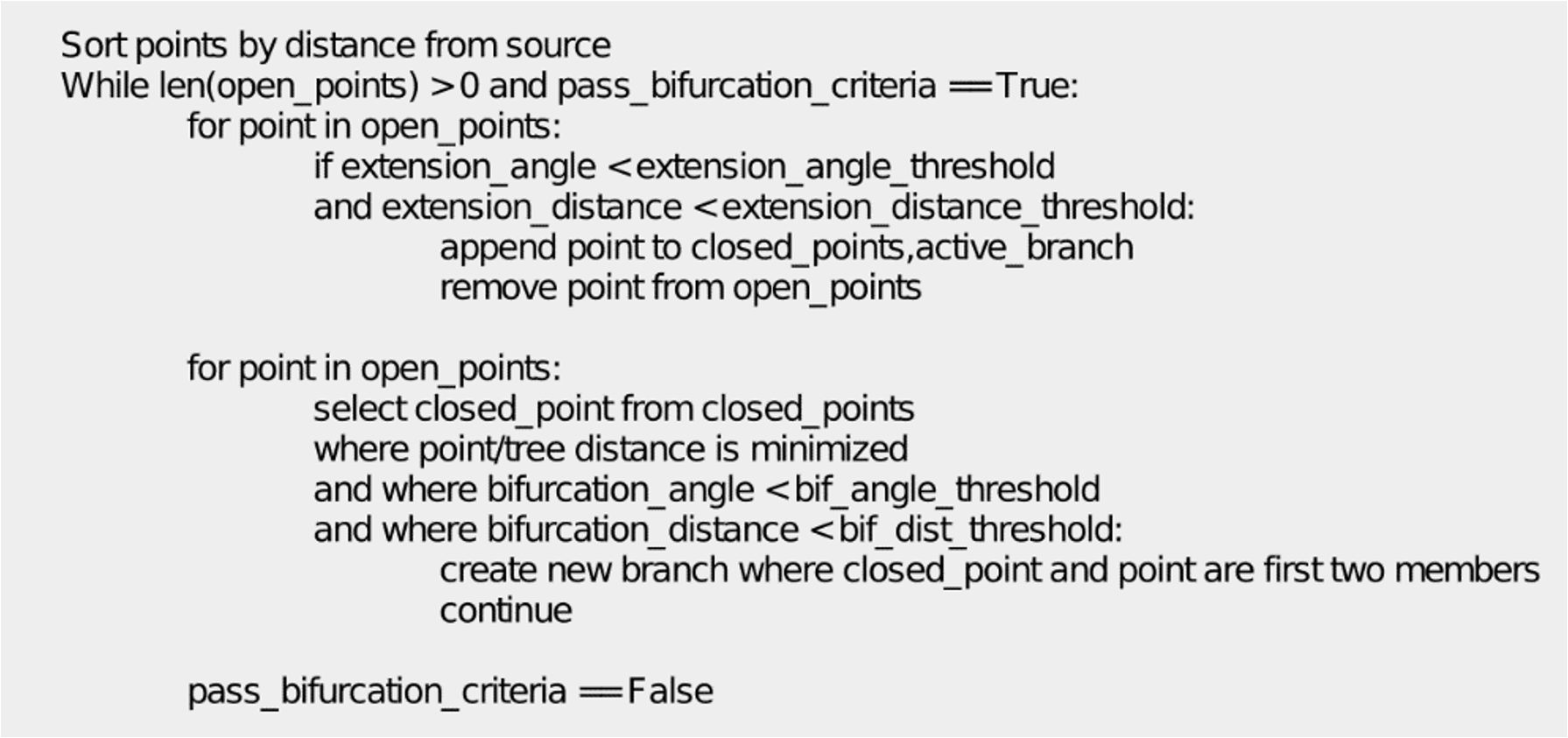

Supplement: FIGURE S1 — Pseudocode of the core algorithm as presented in this study. Other components such as source point reassignment, and topology simplification procedures are executed in series with (before or after) the steps outlined here. [file Image_1.TIF]

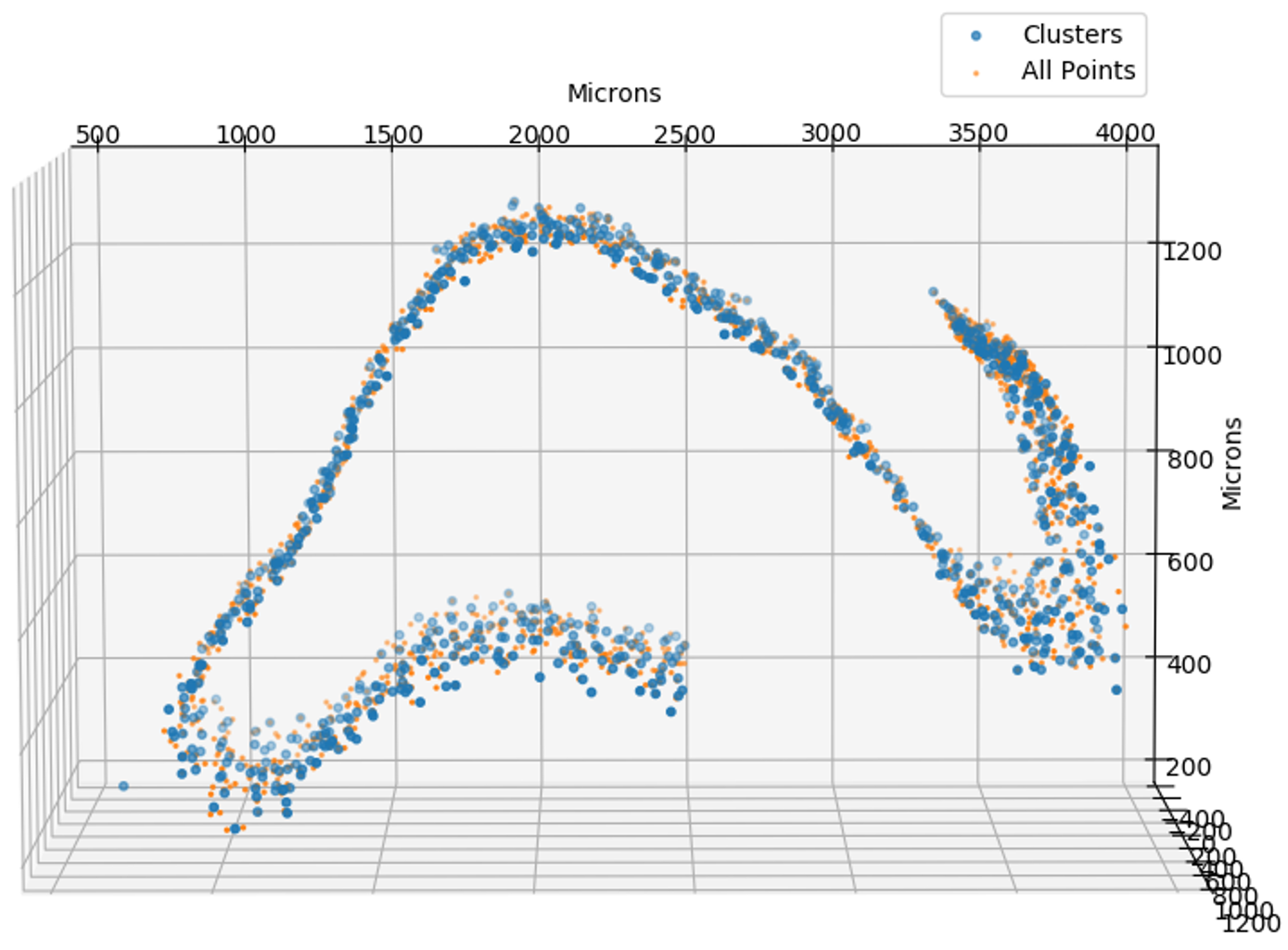

Supplement: FIGURE S2 — Clustering is performed via K-means clustering method. This regularizes the spatial properties of the mesh which will comprise the volume of the arbor and reduces computational complexity of subsequent steps. [file Image_2.TIF]

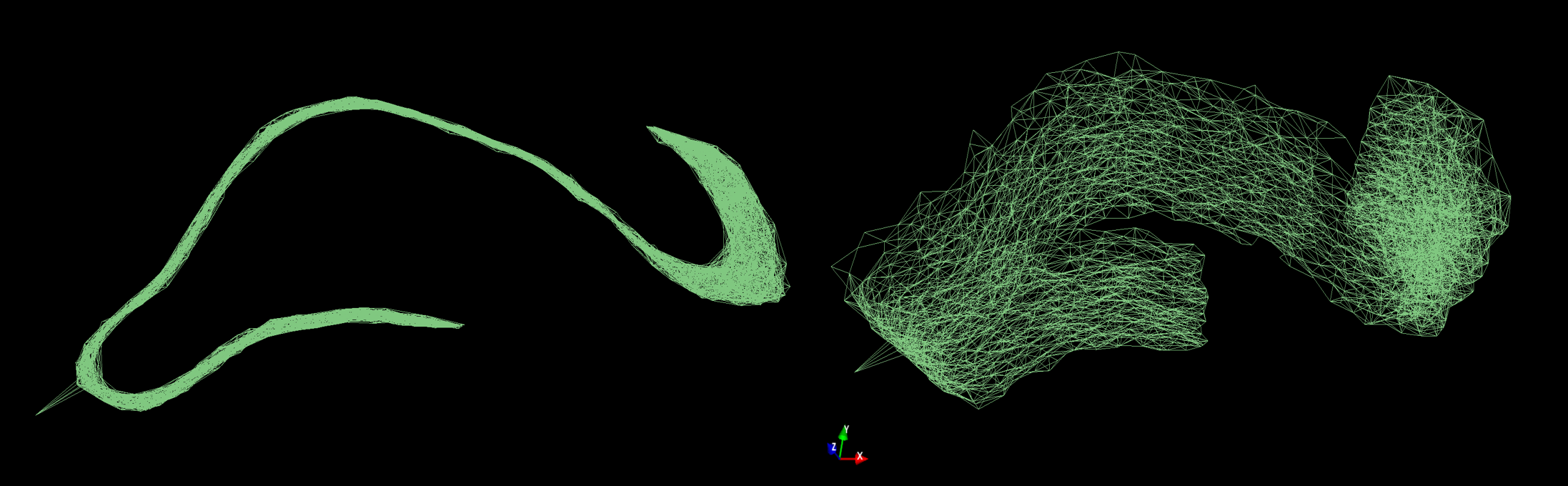

Supplement: FIGURE S3 — Delaunay triangulation to construct a surface from spatially clustered synaptic targets. This yields a network from which ideal paths to potential target zones can be calculated. [file Image_3.TIF]

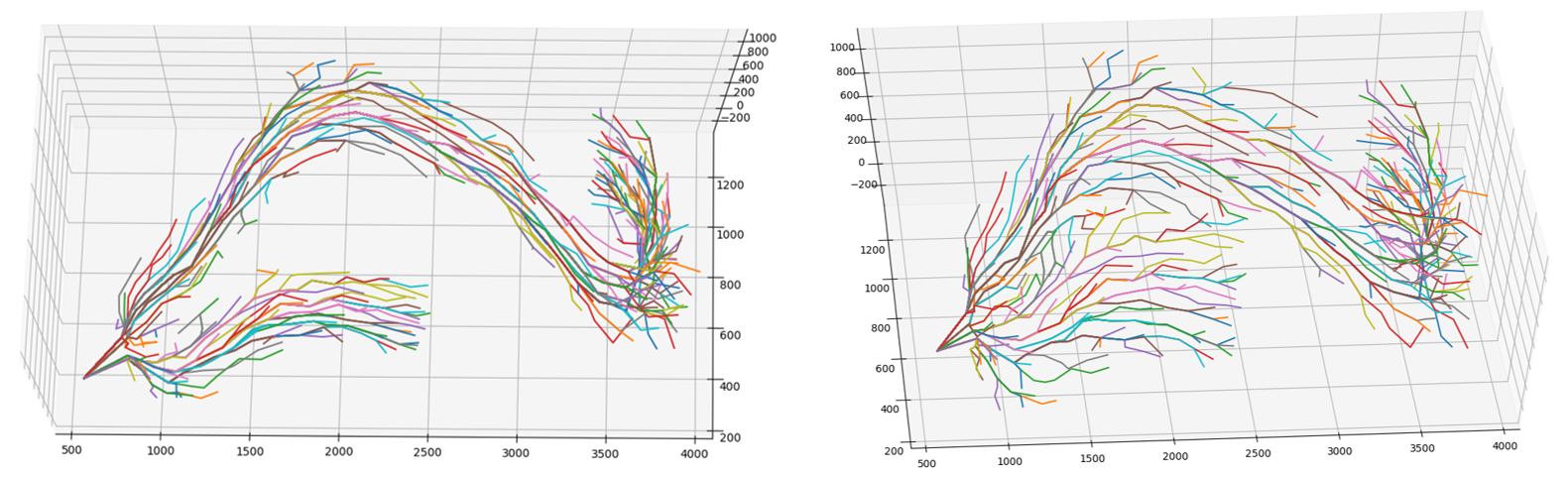

Supplement: FIGURE S4 — Plotting of each “most likely path” between nodes in the Delaunay triangulated network of k-means cluster centers. Paths are constructed via Dijkstra’s algorithm. These paths are provided as inputs to the ROOTS algorithm to loosely guide branching behavior. [file Image_4.TIF]

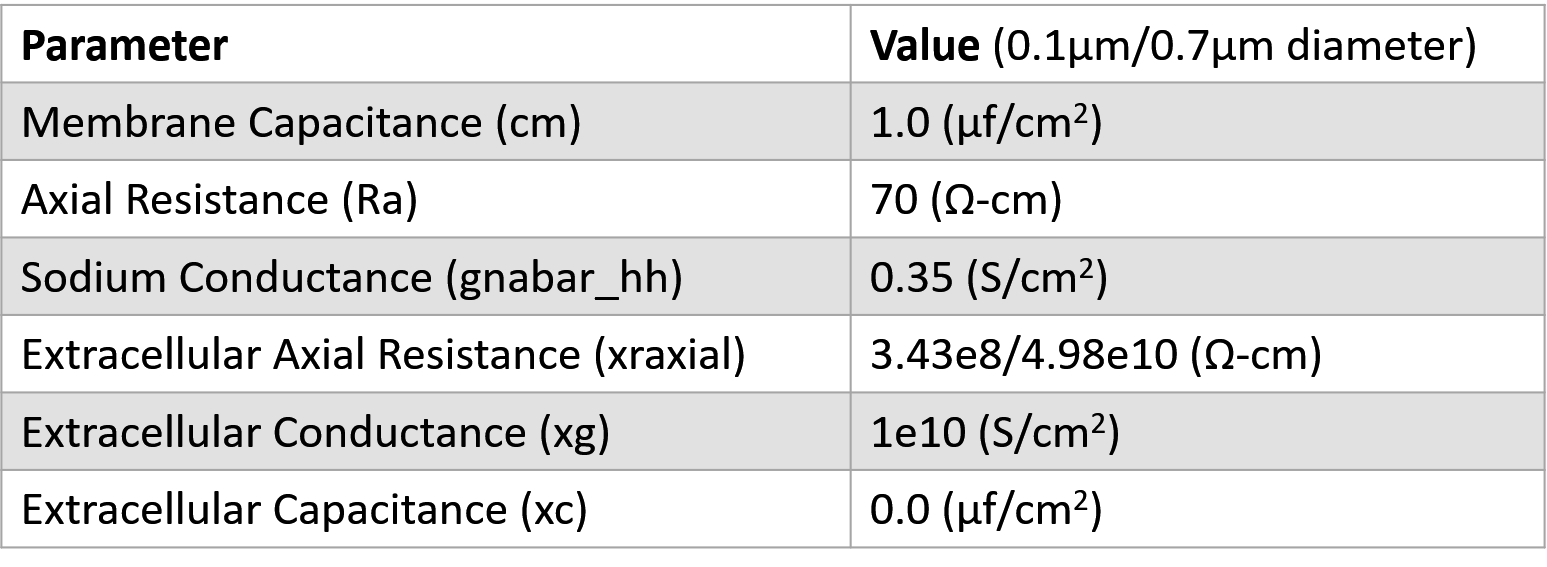

Supplement: FIGURE S5 — A brief table of biophysical parameters used in simulation of NEURON models. The only explicitly varying parameter (those not differing due only to changing compartment areas) was extracellular axial resistance. This followed the pattern implemented in Johnson and McIntyre (2008) to ensure this value was sensitive to fiber diameter. [file Image_5.TIF]

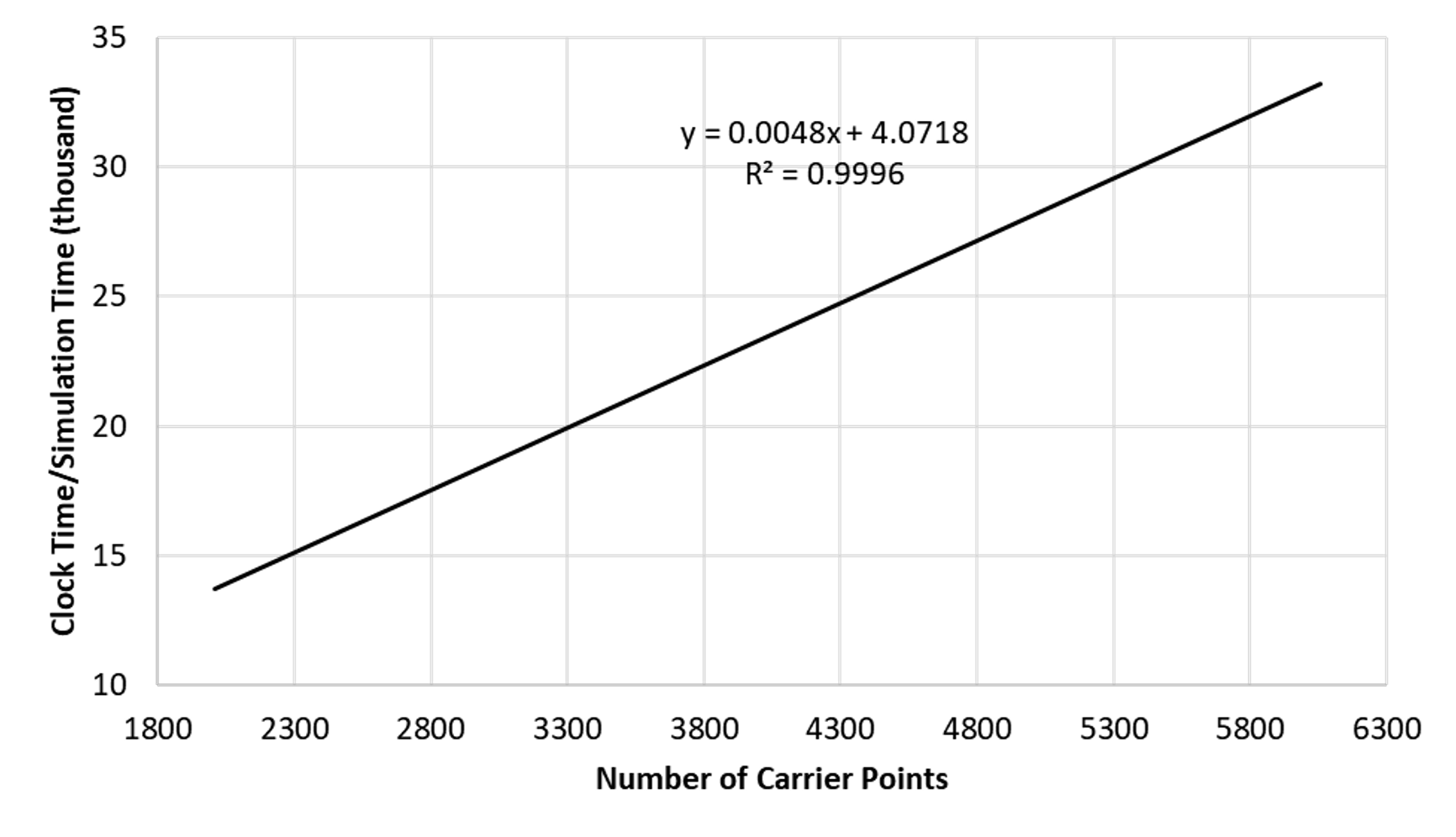

Supplement: FIGURE S6 — Entorhinal cortical axons may form as many as 17,700 synapses with granule cells. However, growing axons with this number of nodes is slow and simulating axons with this complexity is computationally prohibitive when attempting to simulate in situ scale/density tissue models. [file Image_6.TIF]

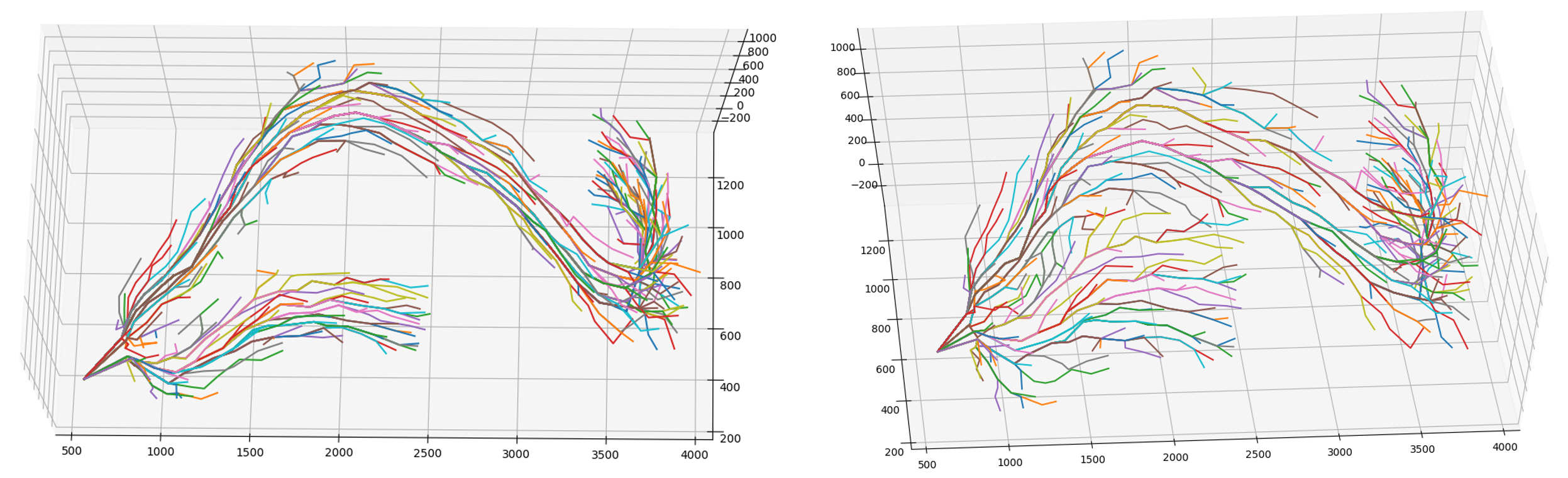

Supplement: FIGURE S7 — Example graph models (red, LEC; teal, MEC) of entorhinal cortical axon terminal fields, generated by the TREES toolbox method. These axons were generated with the addition of a terminal field in CA3/2/1. For very deep folds with small clefts, the TREES MST method has difficulty suppressing trans-cleft connections that short the cortical circuit without inappropriate manipulation of the number and density of targets, further exacerbating the proliferation of terminal branchlets. [file Image_7.TIF]
